# A population study comparing tracheal and lung adenoid cystic carcinoma

**DOI:** 10.1002/cam4.7158

**Published:** 2024-04-04

**Authors:** Yu Gu, Songtao Lai, Yang Wang, Juan Yang, Ping Zhou, Tianxiang Chen

**Affiliations:** ^1^ Department of Radiation Oncology Fudan University Shanghai Cancer Center Shanghai China; ^2^ Department of Oncology, Shanghai Medical College Fudan University Shanghai China; ^3^ Shanghai Clinical Research Center for Radiation Oncology Shanghai China; ^4^ Shanghai Key Laboratory of Radiation Oncology Shanghai China; ^5^ Shanghai Lung Cancer Center, Shanghai Chest Hospital, School of Medicine Shanghai Jiao Tong University Shanghai China; ^6^ Xiaogan Hospital Affiliated to Wuhan University of Science and Technology Xiaogan China; ^7^ Department of Radiotherapy The First Affiliated Hospital of Hainan Medical University Haikou China; ^8^ Department of Thoracic Surgery The First Affiliated Hospital of Wenzhou Medical University Wenzhou China

**Keywords:** adenoid cystic carcinoma, lung and bronchus, tracheal

## Abstract

**Background:**

Thoracic adenoid cystic carcinoma (ACC) is rare, and the differences between tracheal and lung lesions have not been fully understood.

**Methods:**

Patients were identified from a Chinese cancer center (FUSCC) (2005–2022) and the Surveillance, Epidemiology, and End Results (SEER) database (2000–2019). Incidence was calculated and trends were quantified. Clinicopathological features and overall survival (OS) were analyzed. Nomograms predicting OS were constructed.

**Results:**

Totally, 55 tracheal adenoid cystic carcinoma (TACC) and 25 lung and bronchus adenoid cystic carcinoma (LACC) were included in a Chinese cohort, 121 TACC and 162 LACC included in the SEER cohort. There were larger tumor sizes, more lymph nodes and distant metastases for LACC than TACC patients. TACC patients are more likely to get local treatments. Patients with LACC had significantly worse median OS than patients with TACC (SEER cohort: 68.0 months vs. 109.0 months, *p* = 0.001, Chinese cohort: 62.9 months vs. 124.8 months, *p* = 0.061). Age, lymph node metastasis, distant metastasis and local treatment were identified as independent prognostic factors for OS of TACC. Distant metastasis and local treatment were identified for LACC. Specifically, surgery alone or in combination with radiotherapy is crucial for improving survival in both TACC and LACC. Only TACC benefits from radiotherapy alone, while chemotherapy does not improve survival for either. The nomograms constructed using these factors revealed good prognostic accuracy.

**Conclusions:**

LACC is more aggressive and has a worse prognosis than TACC. TACC patients have more opportunities for local treatment, which is important for the prognosis of both TACC and LACC. Nomograms were created for TACC and LACC to aid in personalized survival predictions and clinical decisions.

## 1 INTRODUCTION

Adenoid cystic carcinoma (ACC) is a distinct type of malignant epithelial tumor that usually arises in the salivary glands of the head and neck, whereas thoracic ACC is rare. Due to lack of related prospective multi‐center trials, much remains unknown about the clinicopathologic features, treatment, and prognosis of thoracic ACC. Previous studies on thoracic ACC did not differentiate features between tracheal and lung lesions, but in fact these two ACC categories have different clinical characteristics and prognosis.

ACC is the second most common malignancy of the trachea, accounting for 30%–40% of cases.^1^ Primary ACC arises from the bronchial glands and is more densely distributed in the central bronchi than in the segmental bronchi,^2^ thus the occurrence of ACC in the peripheral lungs is more rare. Among lung and bronchus malignant tumors, ACC accounts for about 0.1%–0.2% of all cases.^3^

Trachea ACC (TACC) is considered to grow slowly, however due to its specific histological features, it may invade extensively inside and outside the tracheal wall. Due to their unique biological behavior and disease sites, complete resection is difficult to achieve, and there is a high probability of local recurrence and distant metastasis, so multi‐disciplinary treatment are required. However, tumors arising of lung show different prognostic patterns and symptoms from that of trachea, and some peripheral lung ACC may originate from sub‐segmental bronchi.^4^ They tend to exhibit rapid growth, locally aggressive behavior, and large tumor volume.^5^

The aim of this study was to compare the difference between TACC and lung and bronchus ACC (LACC). Given the extreme rarity and long survival of thoracic ACC, a sufficiently large sample size and a sufficiently long follow‐up time are usually required for analysis. Therefore, the Surveillance, Epidemiology, and End Results (SEER) database and a Chinese cancer center database were used to compare incidence trend, clinicopathological characteristics and survival outcome, and to establish characteristic prognostic nomograms for TACC and LACC patients. This study mainly emphasizes that thoracic ACC should be treated differently according to different originated tumor sites.

## 2 METHODS

### 2.1 Population cohort

#### 2.1.1 SEER cohort

Clinicopathological data were extracted from a representative patient population with the use of SEER*Stat, version 8.4.0.1 (http://seer.cancer.gov/). SEER 17 Regs Custom Data (with additional treatment fields) from 2000 to 2019 was selected.

Patients were selected for this study according to the following inclusion criteria: (i) Primary ACC were recognized by the International Classification of Disease for Oncology, Third Edition (ICD‐O‐3) code 8200/28200/3. (ii) Site codes were identified as “C33.9 Trachea” and “C34.0–34.9 Lung and bronchus.” (iii) ACC of trachea or lung and bronchus was the only and first primary cancer. (iv) Diagnose by histology.

#### 2.1.2 FUSCC cohort

Between May 2005 and August 2022, patients with primary ACC were identified pathologically from Fudan University Shanghai Cancer Center (FUSCC). ACC was diagnosed according to the World Health Organization classification of thoracic tumors. Patient follow‐up was conducted by telephone and medical records.

### 2.2 Data acquisition and statistical analysis

Patients demographics (age, race, and gender), tumor characteristics (laterality, size, T stage, lymph node metastasis, and distant metastasis), treatment (local treatment [including surgery and radiotherapy] and chemotherapy) and follow‐up data were collected. LACC was staged using the lung cancer tumor node‐metastasis (TNM) staging system in the International Union Against Cancer (8th edition).^6^ Categorical variables were presented as counts and percentages and were compared utilizing chi‐squared tests. Continuous variables were compared using the *t* test.

The incidence of ACC was calculated. Incidence was defined as new cases per 100,000 persons and was age‐adjusted for the 2000 US standard population in million and analyzed with the use of SEER*Stat. Significant changes and trends were analyzed by using Joinpoint Regression Program software.

Reverse Kaplan–Meier method was used for calculating median follow‐up time. Kaplan–Meier analysis was used to determine survival, log‐rank tests was used to compare survival curves. Univariate and multivariate analyses of survival‐related variables were performed using Cox proportional hazards model. Variables were screened by univariate model (*p* < 0.1) and then included in multivariate Cox model to determine independent risk factors for OS in patients with TACC and LACC. The statistical analysis was conducted using IBM SPSS Statistics 22.0.

A nomogram model was established according to the results of multivariate analysis. We used the SEER database as the training cohort and FUSCC database as the external validation cohort. Discrimination was evaluated using the concordance index (C‐index). A higher value of C‐index indicates a higher accuracy. The calibration was conducted by comparing the nomogram predicted probabilities with the actual probabilities. All data were analyzed using R version 3.2.3.

## 3 RESULTS

### 3.1 Incidence trends of TACC and LACC patients

**FIGURE 1:**
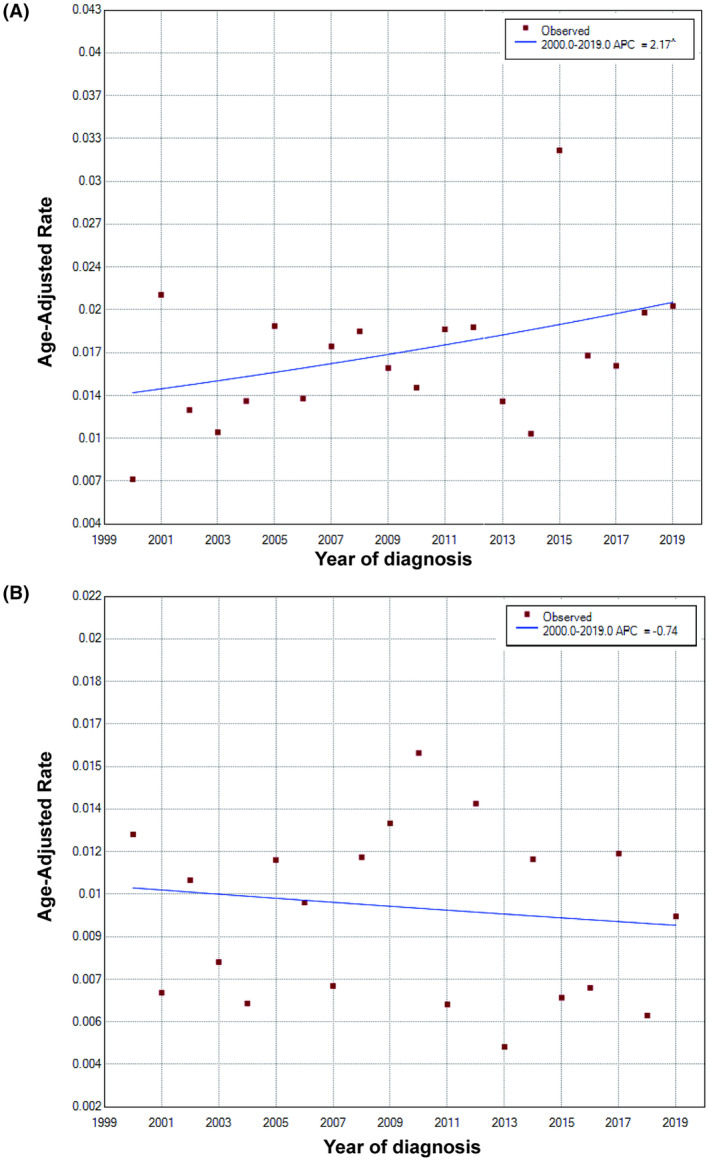
Change in the incidence of primary lung and bronchus adenoid cystic carcinoma (A) and tracheal adenoid cystic carcinoma and (B) patients from 2000 to 2019.

Although both TACC and LACC are rare tumors, the incidence trends for them differ. The age‐adjusted incidence rates of primary LACC was 0.017 per 100, 000. There was an upward trend from 2000 to 2019 for LACC (annual percentage change, APC = 2.17%, *p* = 0.1) (Figure 1A). On the other hand, the age‐adjusted incidence rates of primary TACC was 0.010 per 100,000 in the same period, and showed a downward trend from 2000 to 2009 (APC = − 0.74%, *p* = 0.6) (Figure 1B).

### 3.2 Demographic and clinicopathologic characteristics

Overall, 121 TACC, 162 LACC met the eligibility criteria in the SEER cohort. In FUSCC cohort, a total of 55 TACC and 25 LACC were included. We compared the TACC patients with LACC patients in two cohort, and detailed clinicopathological features were summarized in Table 1.

**TABLE 1:** Characteristics of the study population with primary tracheal adenoid cystic carcinoma (TACC) and lung and bronchus adenoid cystic carcinoma (LACC).

		SEER cohort			FUSCC cohort		
Characteristics		TACC (*N* = 121)	LACC (*N* = 162)	*p*	TACC (*N* = 55)	LACC (*N* = 25)	*p*
Age (year)		54.3 ± 16.4	56.4 ± 14.9	0.261	49.8 ± 13.0	54.4 ± 11.9	0.138
Race	Black	13, 10.7%	19, 11.7%	***0.036***	/	/	/
	White	82, 67.8%	128, 79.0%				
	Other^#^	25, 20.7%	14, 8.6%				
	Unknown	1, 0.8%	1, 0.6%				
Gender	Male	50, 41.3%	80, 49.4%	0.178	25, 45.5%	10, 40.0%	0.649
	Female	71, 58.7%	82, 50.6%		30, 54.5%	15, 60.0%	
Laterality	Left	/	81, 50.0%	/	/	7, 28.0%	/
	Right		75, 46.3%			11, 44.0%	
	Bilateral		1, 0.6%			7, 28.0%	
	Unknown		5, 3.1%			0, 0.0%	
Size	≤3 cm	54, 44.6%	62, 38.3%	0.134	25, 45.5%	5, 20.0%	***0.029***
	>3 cm	39, 32.2%	71, 43.8%		30, 54.5%	20, 80.0%	
	Unknown	28, 23.1%	29, 17.9%		0, 0.0%	0, 0.0%	
T stage	T1	/	45, 27.8%	/	/	6, 24.0%	/
	T2		40, 24.7%			2, 8.0%	
	T3		16, 9.9%			3, 12.0%	
	T4		44, 27.2%			14, 56.0%	
	Tx		17, 10.5%			0, 0.0%	
Lymph node metastasis	N0	85, 70.2%	96, 59.3%	***<0.001***	38, 69.1%	12, 48.0%	0.071
	N+	10, 8.3%	56, 34.6%		17, 30.9%	13, 52.0%	
	N1	/	27, 16.7%	/	/	7, 28.0%	/
	N2		21, 13.0%			6, 24.0%	
	N3		8, 4.9%			0, 0.0%	
	Unknown	26, 21.5%	10, 6.2%		0, 0.0%	0, 0.0%	
Distant metastasis	M0	84, 69.4%	126, 77.8%	***<0.001***	45, 81.8%	11, 44.0%	***0.001***
	M1	11, 9.1%	34, 21.0%		10, 18.2%	14, 56.0%	
	Unknown	26, 21.5%	2, 1.2%		0, 0.0%	0, 0.0%	
Local treatment	None	25, 20.7%	60, 37.0%*	***<0.001***	7, 12.7%	10, 40.0%*	***0.001***
	Surgery only	22, 18.2%	62, 38.3%*		13, 23.6%	11, 44.0%	
	Radiotherapy only	5, 4.1%	3, 1.9%		15, 27.3%	2, 8.0%	
	Radiotherapy and surgery	69, 57.0%	37, 22.8%*		20, 36.4%	2, 8.0%*	
Surgery type	Not performed	/	63, 38.9%	/		12, 48.0%	/
	Partial resection		19, 11.7%			2, 8.0%	
	Lobectomy or more		79, 48.8%			11, 44.0%	
	Unknown		1, 0.6%			0, 0.0%	
Chemotherapy	Not performed/unknown	94, 77.7%	125, 77.2%	0.917	35, 63.6%	10, 40.0%	***0.048***
	Performed	27, 22.3%	37, 22.8%		20, 36.4%	15, 60.0%	

Note: Bold values are indicates as *p* < 0.05.

* Significantly different from TACC (*p* < 0.05) based on Bonferroni corrected *z*‐tests. # The "Other" race category includes Asian, Pacific Islander and Alaska Natives.

* ^#^ The “Other” race category includes Asian, Pacific Islander and Alaska Natives.

LACC patients had larger tumor sizes than TACC patients, more LACC tumors were larger than 3 cm SEER cohort: (43.8% vs. 32.2%, *p* = 0.134) and in FUSCC cohort (80.0% vs. 54.5%, *p* = 0.029). There were more lymph node metastases for LACC patients than TACC patients both in SEER cohort (34.6% vs. 8.3%, *p* < 0.001) and in FUSCC cohort (52.0% vs. 30.9%, *p* = 0.071). In addition, LACC patients had more distant metastases than TACC patients both in SEER cohort (21.0% vs. 9.1%, *p* < 0.001) and in FUSCC cohort (56.0% vs. 18.2%, *p* = 0.001).

In terms of treatment, the proportion of patients not receiving local treatment (including radiotherapy or surgery) were higher for LACC than TACC both in SEER cohort (37.0% vs. 20.7%,*p* < 0.05) and in FUSCC cohort (40.0% vs. 12.7%, *p* < 0.05). More patients with TACC received surgery and perioperative radiotherapy than those with LACC both in SEER cohort (57.0% vs. 22.8%, *p* < 0.05) and in FUSCC cohort (36.4% vs. 8.0%, *p* < 0.05).

In addition, TACC and LACC patients in FUSCC cohort data showed larger tumor size, more lymph node metastases, more distant metastases, and more advanced staging than those in SEER data.

### 3.3 Survival analyses between TACC and LACC

**FIGURE 2:**
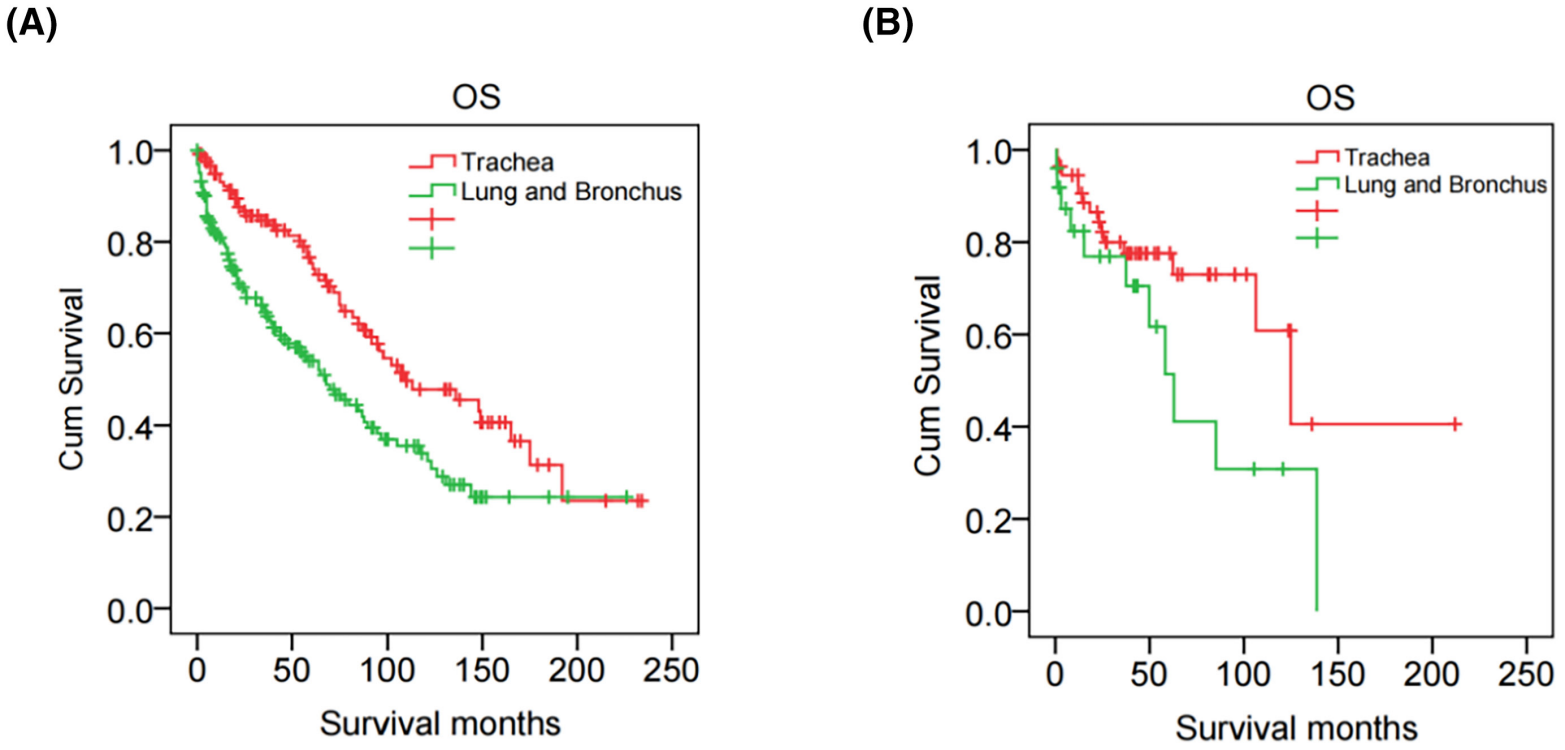
Kaplan–Meier curves of overall survival for patients with tracheal adenoid cystic carcinoma and lung and bronchus adenoid cystic carcinoma in the SEER cohort (A) and FUSCC cohort (B).

The results of the survival analysis for patients with TACC and LACC are presented in Figure 2. Median follow‐up time was 100.0 months (95% CI 85.3–114.7) in SEER cohort. Data from the SEER population showed that overall survival (OS) was significantly lower for LACC patients than that for TACC patients, with a median OS of 68.0 months (95% CI: 48.9–87.1 months) for LACC and 109.0 months (95% CI: 64.0–154.0 months) for TACC (*p* = 0.001) (Figure 2A). After a median follow‐up of 52.6 months (95% CI 41.9–63.2), patients with LACC also had worse median OS than those with TACC in FUSCC cohort (62.9 months, 95% CI: 44.8–80.9 months vs. 124.8 months, 95% CI: 91.7–158.0 months, *p* = 0.061) (Figure 2B). In SEER cohort, the 5‐year OS rates of TACC and LACC were 75.3% and 54.0%, the 10‐year OS rates of TACC and LACC were 47.7% and 33.9% respectively. In FUSCC cohort, the 5‐year OS rates of TACC and LACC were 77.6% and 51.4%, the 10‐year OS rates of TACC and LACC were 60.8% and 30.8%, respectively.

### 3.4 Univariate and multivariate analyses of prognostic factors

The SEER data was assigned as the training cohort. In the training set, variables were screened by univariate model (*p* < 0.1) and then included in multivariate Cox model to determine independent risk factors for OS in patients with TACC and LACC (Table 2 and Table 3). Multivariate analyses showed that age (*p* = 0.001), lymph node metastasis (*p* = 0.022), distant metastasis (*p* = 0.041) and local treatment (*p* = 0.001) were independent prognostic factors for TACC overall survival (Table 2). Distant metastasis (*p =* 0.005) and local treatment (*p* < 0.001) were found to be independent prognostic factors for LACC (Table 3). For LACC, radiotherapy only did not bring survival benefit. Chemotherapy is neither an independent prognostic factor for TACC nor for LACC. Furthermore, for patients who underwent surgery, we analyzed the impact of the surgery type on OS and found no statistical significance, as shown in the Table S1.

**TABLE 2:** Univariate and multivariate Cox proportional hazards analysis of overall survival of tracheal adenoid cystic carcinoma of SEER cohort.

		Univariate analysis			Multivariate analysis		
Characteristics	Level	HR	95% CI	*p* value	HR	95% CI	*p* value
Age		1.024	1.003–1.044	**0.021**	1.064	1.025–1.104	**0.001**
Race				0.641	–		
	Black						
	White						
	Other						
Gender				0.245	–		
	Male						
	Female						
Size				0.057			0.294
	≤3 cm						
	>3 cm						
Lymph node metastasis				**0.036**			**0.022**
	N0	–	–	–			
	N1	2.588	1.062–6.308		5.249	1.273–21.637	
Distant metastasis				**0.030**			**0.041**
	No	–	–	–			
	Yes	2.725	1.105–6.722		7.475	1.087–51.385	
Local treatment				**<0.001**			**0.001**
	None	–	–	–			
	Surgery only	0.183	0.072–0.470	**<0.001**	**0.048**	0.007–0.322	**0.002**
	Radiotherapy only	0.196	0.026–1.477	0.114	**0.024**	0.002–0.354	**0.007**
	Radiotherapy and surgery	0.275	0.144–0.523	**<0.001**	0.160	0.053–0.481	**0.001**
Chemotherapy				**0.043**			0.307
	Not performed/unknown	–	–	–			
	Performed	1.985	1.023–3.851				

Note: Bold values are indicates as *p* < 0.05.

**TABLE 3:** Univariate and multivariate Cox proportional hazards analysis of overall survival of lung and bronchus adenoid cystic carcinoma of SEER cohort.

		Univariate analysis			Multivariate analysis		
Characteristics	Level	HR	95% CI	*p* value	HR	95% CI	*p* value
Age		1.021	1.006–1.037	0.008			0.263
Race				0.177	–		
	Black						
	White						
	Other						
Gender				0.656	–		
	Male						
	Female						
Laterality				0.003			0.242
	Left	–	–	–			
	Right	1.776	1.141–2.765	0.011			
	Bilateral	53.549	5.883–487.416	<0.001			
T stage				0.009			0.574
	T1	–	–	–			
	T2	1.052	0.560–1.974	0.875			
	T3	1.129	0.476–2.673	0.783			
	T4	2.497	1.381–4.515	0.002			
Lymph node metastasis				0.012			0.607
	N0	–	–	–			
	N1	0.768	0.397–1.485	0.432			
	N2	1.924	1.073–3.453	0.028			
	N3	3.316	1.387–7.929	0.007			
Distant metastasis				<0.001			**0.005**
	No	–	–	–	–	–	–
	Yes	4.300	2.667–6.931		2.339	1.298–4.212	
Local treatment				<0.001			**<0.001**
	None	–	–	–	–	–	–
	Surgery only	0.191	0.111–0.329	<0.001	0.240	0.128–0.447	**<0.001**
	Radiotherapy only	1.469	0.454–4.750	0.521	1.512	0.351–6.511	0.579
	Radiotherapy and surgery	0.152	0.078–0.299	<0.001	0.199	0.096–0.412	**<0.001**
Chemotherapy				0.028			0.922
	Not performed/Unknown	–	–	–			
	Performed	1.695	1.058–2.716				

Note: Bold values are indicates as *p* < 0.05.

### 3.5 Construction and validation of prognostic nomograms

**FIGURE 3:**
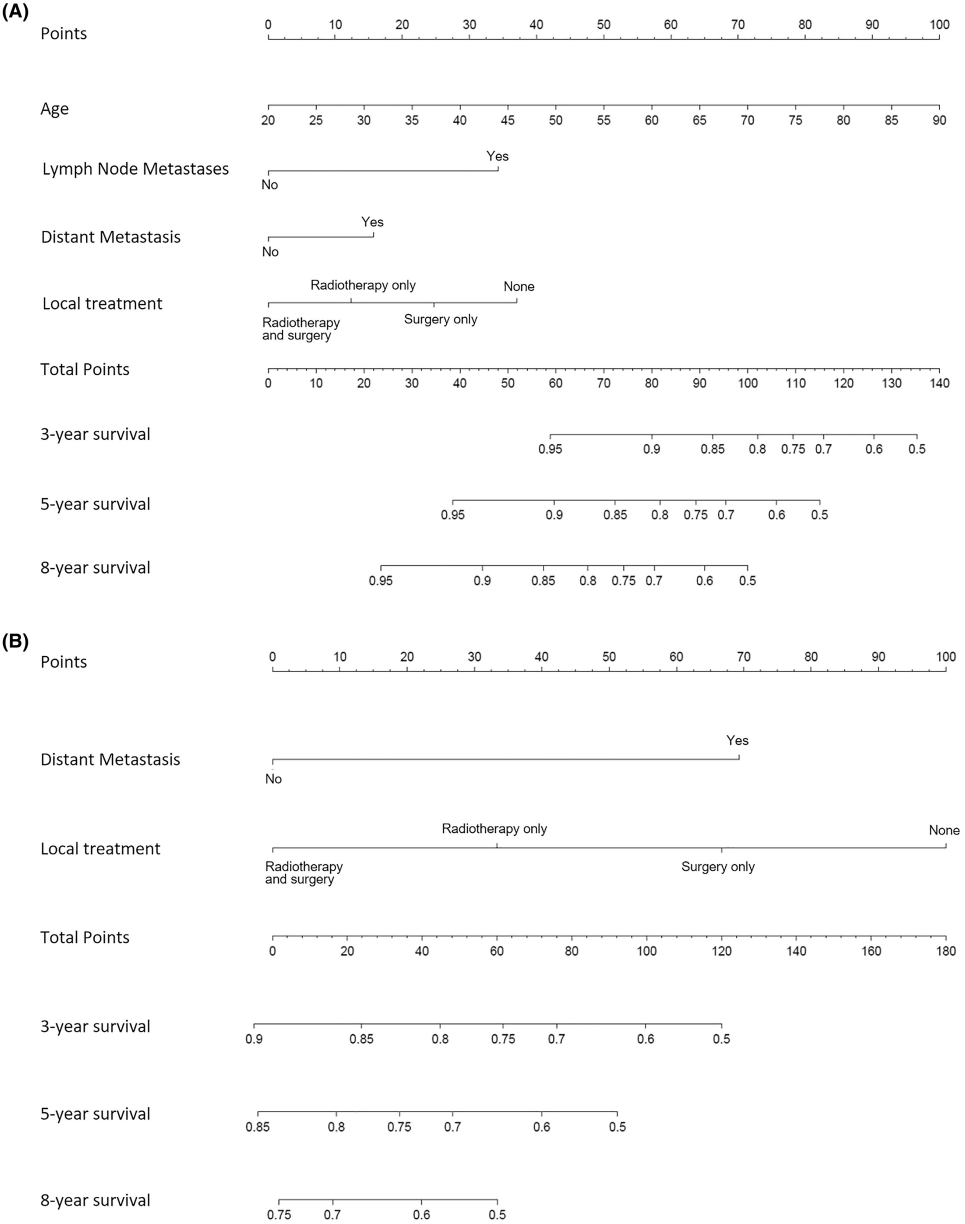
Nomogram predicting the 3‐year, 5‐year and 8‐year overall survival for patients with tracheal adenoid cystic carcinoma (A) and lung and bronchus adenoid cystic carcinoma (B).

**FIGURE 4:**
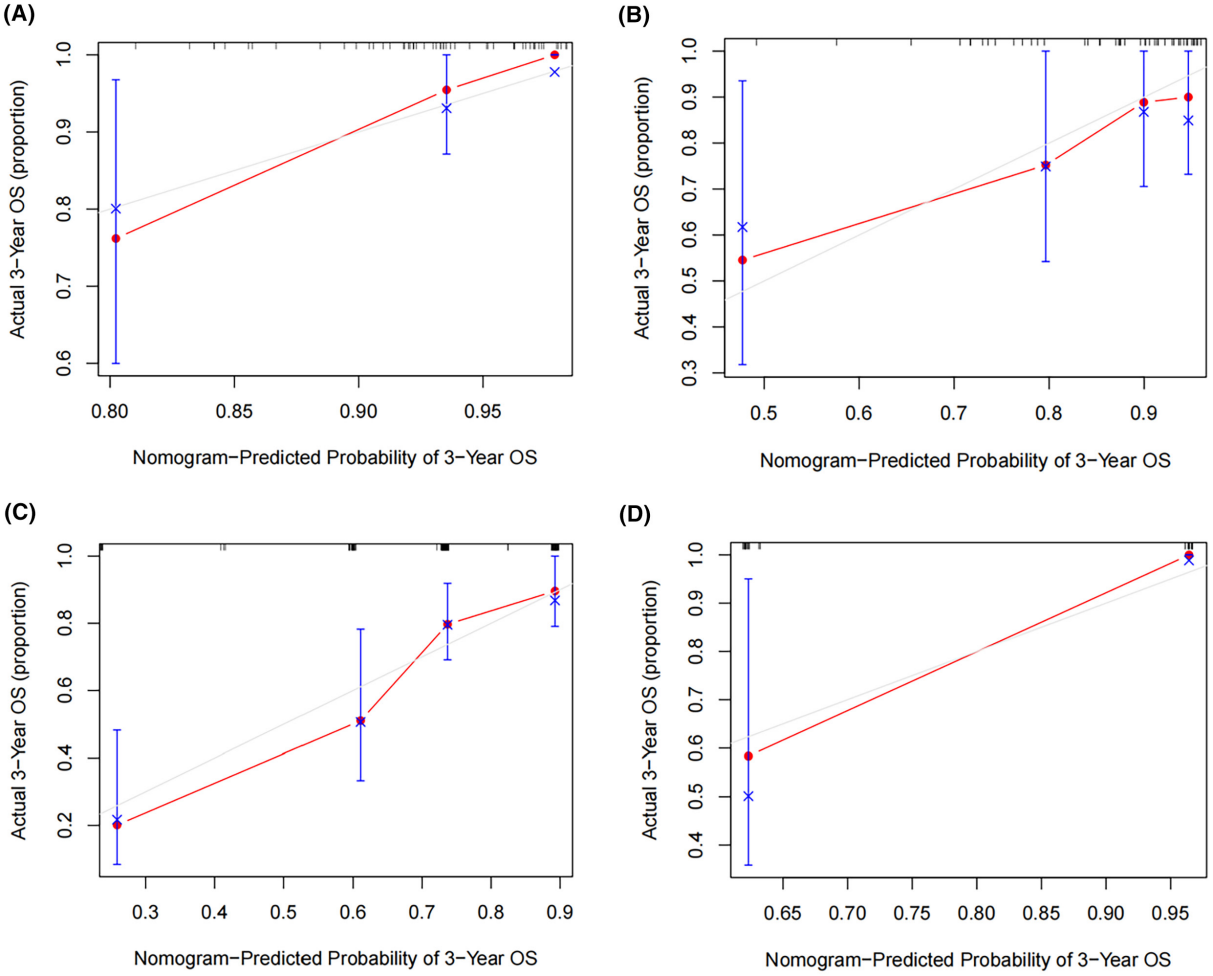
Calibration curves for predicting 3‐year overall survival (OS) nomogram in patients. (A) OS for patients with tracheal adenoid cystic carcinoma (TACC) in the training cohort. (B) OS for patients with TACC in the validation cohort. (C) OS for patients with lung and bronchus adenoid cystic carcinoma (LACC) in the training cohort. (D) OS patients with LACC in the validation cohort.

We developed prognostic nomograms for OS at 3, 5, and 8 years for patients with TACC (Figure 3A) and LACC (Figure 3B) in the training set based on the results of multivariate Cox regression analysis. The nomograms of TACC and LACC are different. Each variable has a score on the scale, and by summing the total scores in the bottom scale, the nomogram can predict 3‐, 5‐, and 8‐year OS for individual patients. The constructed nomogram showed a clear prognosis, the C‐index for TACC was 0.766, for LACC was 0.724. In the validation cohort, the nomogram still showed nice discrimination, the C‐index for TACC was 0.766, for LACC was 0.734. Figure 4 presents the calibration plots which show the predicted OS probabilities for training and validation sets compared with the actual observations.

## 4 DISCUSSION

Primary thoracic ACC is very rare, and long‐term follow‐up is required due to the long survival time, thus most studies so far are small series or single case reports. Therefore, the clinicopathological features, treatment options, and prognostic outcomes of ACC are not well defined. Primary ACC is thought to arise from ductal and myoepithelial cells of the submucosal glands.^4^ The glands are more densely distributed in the central bronchi than in the segmental bronchi. Peripheral lung ACC may arise from subsegmental bronchi, where the glandular distribution is more sparse. The incidence of ACC from the peripheral lung is relatively low and may be closely related to the distribution of glandular cells.^7^, ^8^ Although TACC and LACC have similar histological features, they differ in incidence, clinical features, prognosis, and treatment, and should be treated differently. To the best of our knowledge, this is the first study to systematically and comprehensively compare different tumor location and its relationship with clinical features, treatment modality, and prognosis for patients with thoracic ACC.

First, the incidence trends of TACC and LACC are different. From 2000 to 2019, LACC showed an upward trend, while the incidence of primary TACC showed a downward trend during the same period. Because the incidence was very low, these trends were not statistically significant and further observations were required. The clinical characteristics of our data with TACC patients were consistent with previously reported cases, both in terms of age and sex ratio.^9^, ^10^, ^11^, ^12^

ACC of the trachea and lung is generally considered a low‐grade malignancy and has a better prognosis than other pathological types.^10^, ^13^ In fact, there are significant differences in the prognosis of TACC and LACC, our study found that the survival rate of TACC patients was much higher than that of LACC patients. And larger tumor size, more lymph node metastasis and distant metastasis were found in LACC. Literature suggests that regional nodal involvement in TACC is uncommon.^14^ Previous studies have shown that for thoracic ACC, the rate of lymph node metastasis and distant metastasis is higher than that of other salivary gland tumors, but this study did not separate TACC and LACC.^10^ Zhao et al. found that ACC of bronchus origin was associated with higher lymph node involvement and decreased disease‐free survival compared with TACC.^15^ Subgroup analysis of a retrospective study showed that ACC of bronchial origin was a significant predictor of lymph node metastasis and had a worse prognosis compared with tracheal ACC.^16^ This suggests that ACC in the bronchi behave more aggressively than ACC in the trachea. This could be related to the fact that primary tracheal tumors are more likely to present symptoms and be detected earlier.^15^ In addition, these findings confirm that differences in the location of origin between trachea and bronchi may underlie the heterogeneity of TACC and LACC, which may have important clinical implications, future studies on biological behavior are warranted.

Furthermore, due to the rarity of thoracic ACC, data on the treatment outcomes of various therapies are relatively limited. There is little evidence on how ACC should be treated and it is often based on experience with ACC in the head and neck. From the perspective of treatment options, there are also differences between TACC and LACC. More TACC patients receive perioperative radiotherapy and surgery compared to LACC, and this approach is crucial for improving the overall survival. Because this type of tumor tends to grow extensively along the tracheobronchial tree, TACC is difficult to operate, often requiring resection of a longer trachea, and laryngotracheotomy may be considered in some case.^17^ In addition, surgery for safer anastomotic tension tends to result in higher rate of positive margins.^15^, ^18^ Although some case series showed that surgical resection with negative margins was an important prognostic factor, R1 resection followed by radiotherapy resulted in comparable long‐term survival to TACC patients with R0 resection.^11^, ^19^, ^20^, ^21^, ^22^ For LACC patients, fewer receive local treatment compared to TACC, which may be related to the more aggressive biological behavior and later staging of LACC. Among local treatment options, only radiotherapy is less important, while surgery plays a more significant role. Moreover, consistent with previous finding, chemotherapy does not provide survival benefits for either of these cancers.^23^

There are still some limitations in our study. First, some detailed clinical information was not represented in the SEER database, such as surgical margin, smoking history, etc., and descriptions regarding chemotherapy were limited to “yes” and “no/unknown,” potential mis‐coding resulting in certain information inaccuracy. Second, this is a retrospective analysis study, and the conclusions drawn still need to be verified by prospective studies. Also, studies of rare tumors inevitably introduce some statistical bias. However, to our knowledge, this is the first report that systematically compares TACC and LACC. Although our study was not a prospective randomized controlled study, it was population‐based and reflected real‐world clinical characteristics, practices, and outcomes which made up for its limitations to some extent. This study allowed us to distinguish the characteristics and treatment strategies of the two ACCs, and to establish two different prediction models, which still need more research data to verify in future.

## 5 CONCLUSIONS

In conclusion, this population‐based study showed that LACC is more aggressive and has a worse prognosis than TACC. TACC patients are more likely to get local treatments like surgery combined with radiotherapy compared to LACC. These treatments are crucial for improving survival in both TACC and LACC. Only TACC benefits from radiotherapy alone, while chemotherapy doesn't improve survival for either. We developed and validated two different nomograms for predicting the survival of TACC and LACC patients.

## AUTHOR CONTRIBUTIONS

**Yu Gu:** Conceptualization (equal); data curation (lead); writing – original draft (lead). **Songtao Lai:** Data curation (equal); formal analysis (equal). **Yang Wang:** Formal analysis (equal); methodology (equal). **Juan Yang:** Formal analysis (equal); validation (equal). **Ping Zhou:** Investigation (equal); writing – review and editing (equal). **Tianxiang Chen:** Conceptualization (equal); funding acquisition (equal); writing – review and editing (lead).

## CONFLICT OF INTEREST STATEMENT

The authors declare no competing interests.

## Supporting information


Table S1.

## Data Availability

The datasets used during the present study are available from the corresponding author upon reasonable request.
