# Supplementary material for: A population study comparing tracheal and lung adenoid cystic carcinoma
Source: Cancer Med. 2024 Apr 4;13(7):e7158. doi: 10.1002/cam4.7158 (PMC10993707; doi:10.1002/cam4.7158)
Supplement: Supplementary file 1 — Table S1. [file CAM4-13-e7158-s001.docx]

Supplementary Table1 Univariate and Multivariate Cox Proportional Hazards Analysis of Overall survival(OS) of lung and bronchus adenoid cystic carcinoma of SEER cohort.

|  |  | SEER LACC | FUSCC LACC |
| --- | --- | --- | --- |
|  | Level | *p* value | *p* value |
| Surgery Type | Partial Resection | 0.704 | 0.433 |
|  | Lobectomy or more |  |  |
